# Synchronous Driving Method for Stitching Pixel Arrays Based on an Adaptive Correction Technique

**DOI:** 10.3390/s24061886

**Published:** 2024-03-15

**Authors:** Suiyang Liu, Zhongjie Guo, Xinqi Cheng, Ruiming Xu, Ningmei Yu

**Affiliations:** Department of Electronic Engineering, Xi’an University of Technology, No. 5, Jinhua South Road, Xi’an 710048, China; syliu@stu.xaut.edu.cn (S.L.); xqcheng@stu.xaut.edu.cn (X.C.); rmxu@stu.xaut.edu.cn (R.X.); yunm@xaut.edu.cn (N.Y.)

**Keywords:** stitching CMOS image sensor, row driving, delay detection

## Abstract

With the application of stitching technology in large-pixel-array CMOS image sensors, the problem of non-synchronized output signals from pixel array bilateral driver circuits has become progressively more serious and has led to the DC perforation of bilateral driver circuits, while conventional clock tree synchronization design methodology does not apply to stitching technology. Therefore, this paper analyses reasons for the inconsistency in the output signals of bilateral driving circuits and proposes a synchronous driving method applicable to stitching pixel arrays based on the idea of on-chip output signal delay detection and calibration. This method detects and corrects the non-synchrony of the row driver output signals on both sides according to changes in the operating environment of the chip. This method is characterized by a simple structure and high reliability. Finally, based on the 55 nm stitching process, simulations are carried out in a CMOS image sensor with a chip area of 77 mm × 84 mm to verify that this method is feasible. This large image sensor with a 150 M pixel array has a frame rate of over 10 FPS.

## 1. Introduction

The image sensor is an important part of a camera. At present, commonly used image sensors are mainly divided into CMOS image sensors (CISs) and CCD image sensors. CMOS image sensors have gained a lot of attention due to their low power consumption and high frame rate. In the field of aerial mapping, astronomical observation, and other applications, high-resolution CMOS image sensors are needed to obtain clearer object details [1,2,3,4]. In 2D CMOS image sensors, a high resolution implies a larger pixel array and a larger chip area. To provide a short settling time for the signals transmitted on the pixel array’s row control bus, row driver circuits are generally designed on both sides of the pixel array [5,6,7].

Thanks to its rapid development, stitching technology [8] provides manufacturing support for the research, design, and implementation of ultra-large array CISs. The stitching process, by using as many repeating stitching blocks as possible, enables the processing of ultra-large pixel array image sensors on small-sized wafers with the advantage of cost savings. Since only one timing control module is needed for the entire chip, there is no need to duplicate it. Therefore, placing the timing control module on one corner of the chip is a commonly adopted strategy in very large pixel array image sensors using the stitching process. In CISs with very large arrays, due to the wide lateral width of the pixel array, when the timing control module located on one side of the chip transmits signals to the row driver module on the other side, it needs to pass through a long-distance transmission wire. The large parasitic resistance and capacitance introduced by the long-distance transmission wire lead to a significant delay in the signal transmission process. However, the transmission distance between the timing control module and the left row driver module is relatively short, and the delay caused by short-distance transmission is relatively small, which leads to a large difference in the delay times of the signals received by the left and right row driver modules, resulting in inconsistency. The inconsistency of the row driver module on both sides will lead to inconsistency in the output signal of the row driver module, which has a great impact on the frame rate of the large-array CMOS image sensor and the symmetry of the drive signal.

In the design of digital IC back-ends, the clock tree technique is usually used to solve this inconsistency problem [9,10]. However, in the process of designing and manufacturing large-pixel-array CMOS image sensors, stitching technology is used to improve the consistency of the column readout circuit and reduce the manufacturing cost, so the clock tree technique cannot synchronize bilateral row driver signals. In Reference [11], the instability of the clock generation circuit is eliminated through a synchronous reset scheme, which ensures the reliable synchronization of sampled and anti-serial signals between each channel. In References [12,13], a column-parallel digital delay-locked loop with dual clock trees is used to reduce the clock skew of the gated clock. In Reference [14], the timing skew is detected in the digital domain through a correlation-based algorithm and minimized by adjusting digitally controlled delay lines. In Reference [15], the reason why clock tree technology cannot solve this problem is explained in detail, and a delay-locked loop (DLL) design is proposed to improve the synchrony of both row driver signals. However, one DLL can only be used for one row logic signal; when there are multiple row logic signals to be synchronized, multiple delay-locked loops are required to work at the same time. In this way, more area and power consumption losses are caused.

**Figure 1:**
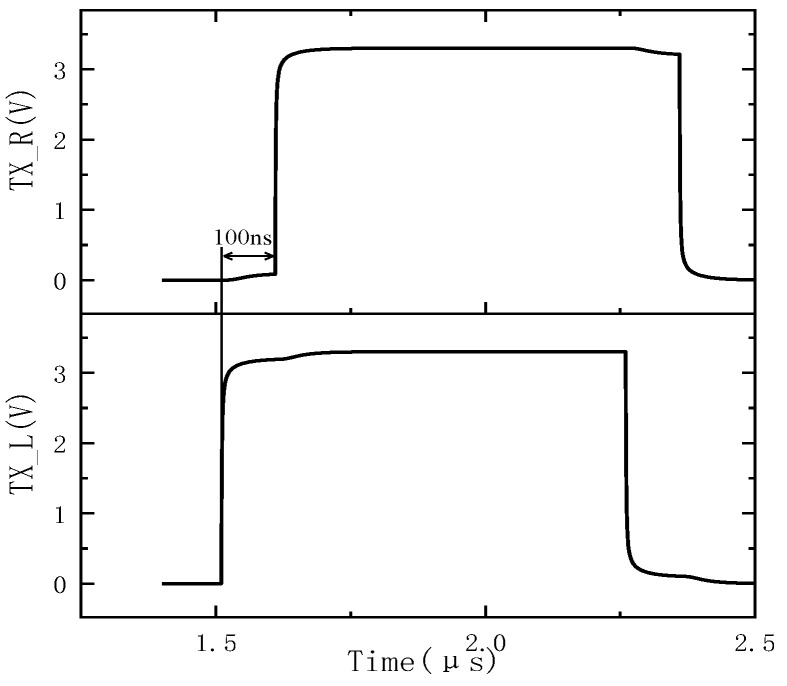
Inconsistent left and right row driver output TX signals.

For image sensors with large pixel arrays, there are many long signal lines. For example, horizontally, there is a signal line to control pixels, and vertically, there is an output signal line of pixels. These signal lines, which are several centimeters long, have a large metal wire parasitic capacitance and metal wire parasitic resistance. As a result, the signal settling time is long, limiting the readout speed of the image sensor. This makes the frame rate of the very-large-pixel-array image sensor only a few frames per second [7,16,17]. Using the TX signals on the left and right sides of the pixel array as an example, inconsistencies in the row driver output signals on both sides of the pixel array will affect the frame rate. As shown in Figure 1, without a compensation technique, the TX signal is transmitted to the right side with a delay of 100 ns compared to the left. Then, the effective pulse width of the signal must be much greater than 100 ns. With the compensation technique, the reach of the TX signal to the right is consistent with the left, and the effective pulse width of the signal can be set shorter. Eventually, the time to read out a row of signals can also be set shorter, and the frame rate of the image sensor will increase accordingly. This illustrates that if the output signals of the drivers on both sides are identical, it will help reduce the output time of the image sensor and increase the frame rate.

Compensation techniques to improve the consistency of row driver output signals help enhance image quality. Since the signals transmitted by the row driver are all critical control signals for the pixel, if the consistency of these signals cannot be guaranteed, at the most basic level, there may be differences in the exposure time of each pixel. This will produce streaks or changes in light and dark on the image output from the image sensor, directly affecting the image quality of the image sensor [5].

This paper is structured as follows. Section 1 presents that in large-pixel-array image sensors using a stitching process, there is a problem with inconsistency in the input signals of the row drivers on both sides. Section 2 describes the traditional approach to solving the problem of inconsistent left and right row drivers, which cannot be applied to the stitching process. In Section 3, a row-driven adaptive correction technique that can be applied to the stitching process is proposed through a theoretical analysis. Section 4 describes the physical implementation of the adaptive correction technique. Section 5 shows the results of the simulation and analyzes them. Finally, conclusions are given in Section 6.

## 2. Application and Restriction of Stitching Technology in Design

**Figure 2:**
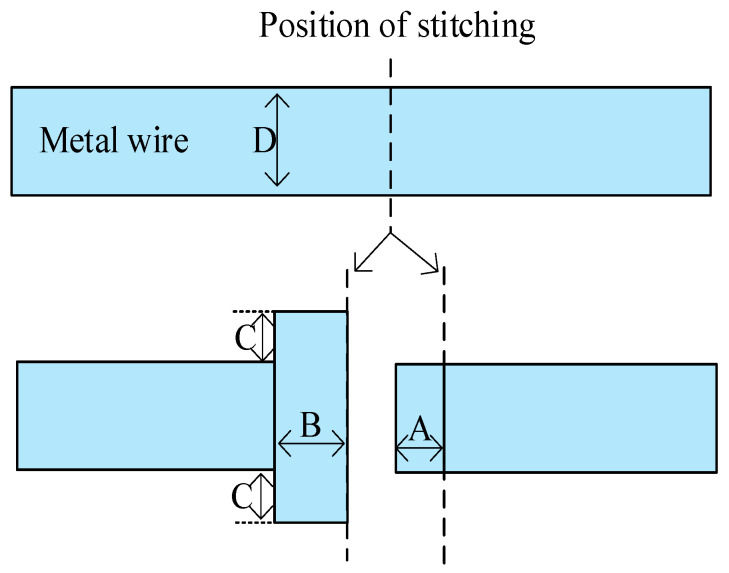
Stitching method for metal wire.

Including the peripheral circuit, the overall CIS size of 12,288 × 12,288 pixels (77 mm × 84 mm) is significantly larger than the typical mask plate size (26 mm × 33 mm). Currently, the only feasible way to produce such chips is to take advantage of stitching technology, which allows different parts of the chip to be repeated on the wafer with very high accuracy. In the stitching process, the active region, well, poly, and metal wire can be stitched as long as the stitching rules are satisfied. The stitching method of the metal wire is shown in Figure 2. In the stitching process, D is the width of the wire. For the wire to be stitched, one end extends a hammer range, which is beyond the stitching boundary for the length of B, and both sides are wider than the wire for the length of C to ensure a certain fault tolerance range during stitching, and the other end extends beyond the stitching boundary for the length of A, which is overlapped with the hammer region during stitching to improve the success rate of stitching. In the design of the layout, it is necessary to design a stitching marking layer to mark the layers that need to be stitched.

**Figure 3:**
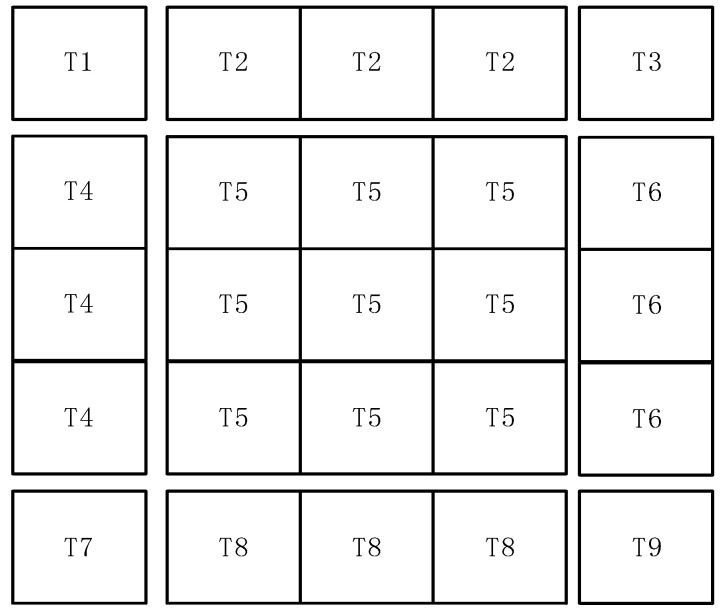
Showing a 150 M pixel scale CIS layout stitching architecture.

**Figure 4:**
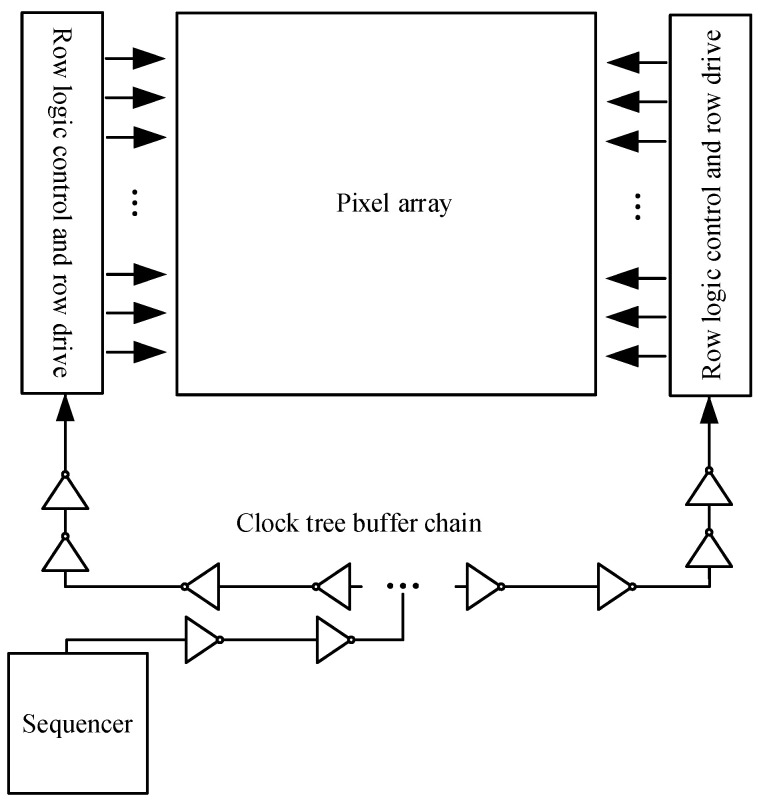
Clock tree technology.

The CIS circuit has high repeatability and is a natural place for the application of stitching technology. The repeated pixel array is the central part, and the readout circuit and pins form the peripheral part. The 12,288 × 12,288 CIS is created by stitching a 3 × 3 pixel array on one wafer. The overall layout of the CIS is shown in Figure 3, where T2 is the tail current source of 4096 columns of pixels, T1 is the DAC module, T3 is the power filter capacitor, T4 and T6 are the row driver modules of 4096 rows on the left and right sides, T5 is a pixel array of 4096 × 4096, T7 produces modules for the sequence, T8 is the readout circuit of column 4096, and T9 is the regulated power supply and reference generation module. Since the three T8 modules are the same, only one mask version of T8 needs to be fabricated, and the mask version is then multiplexed using the stitching process, which can not only ensure the consistency of the readout circuit but also reduces the manufacturing cost. However, the clock tree technique, like the one shown in Figure 4, is not applicable to stitching CISs because the adoption of this technique requires the manufacture of three different T8 mask plates, which reduces the consistency of the readout circuit and increases the tape-out cost.

Given the above problems, this paper proposes an adaptive correction method based on delay detection and accurate compensation technology which can be applied to the stitching process, has high reliability, and can ensure that the bilateral row drive signal has high synchronization.

## 3. Theoretical Analysis

**Figure 5:**
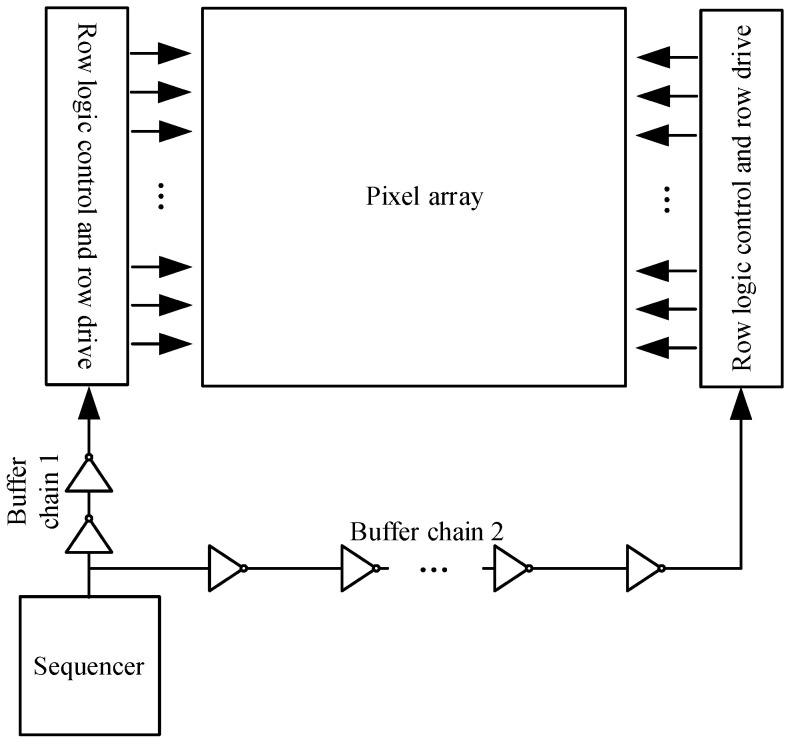
The transmission path of the bilateral row driver signal.

As shown in Figure 5, the control signal issued by the sequencer reaches the row driver module through a buffer chain, and a delay time T_d_ is generated. The buffer chain is composed of a certain number of buffers in series. This approach necessarily leads to different synchronizations of the bilateral row drive signals.

**Figure 6:**
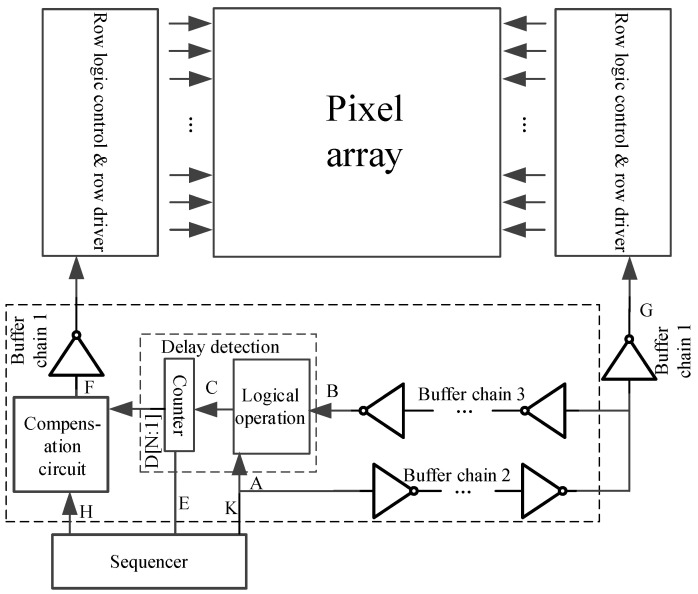
Delay detection and compensation technology structure.

A scheme of the adaptive correction technique for the bilateral row driving signal of a CIS for a large array is shown in Figure 6.

The circuit is mainly divided into two parts: a delay detection circuit and a compensation circuit. The delay detection circuit detects the total delay time of buffer chain 2 and buffer chain 3 and converts the total delay time into the counter value. Then, half of the counter count value is configured into the compensation circuit so that the delay time generated by the compensation circuit is equal to the delay time generated by buffer chain 2. Therefore, in the layout design, it is necessary to ensure that the parasitic environment, the number of buffers inserted, and the wire widths of buffer chain 2 and buffer chain 3 are the same, that is, the delay times of buffer chain 2 and buffer chain 3 are the same. After the delay detection is completed, when the logic signal is output by the sequencer, the logic signal reaches the input terminal G of the right row driver through buffer chain 2 and reaches the input terminal F of the left row driver through the compensation circuit. Therefore, the synchronization of the row drivers on both sides depends on how well the delay time of buffer chain 2 and the delay time of the compensation circuit match. The delay time of the compensation circuit is restricted by the precision of the delay detection circuit.

The delay time of buffer chain 2 can be expressed as

(1) $$T_{d2}\;=\;(T_{\mathrm{DTD}}\pm 1\;\mathrm{clk})/2\;=\;T_{\mathrm{DC}}$$

Here, T_DTD_ is the delay time detected by the delay detection circuit, clk is the clock period, and T_DC_ is the delay time of the compensation circuit. The value of T_DTD_ is generated by the counter, which has an error of less than 1 clk. Therefore, this method has a time residual whose value is 1/2 clk. Defining the compensation accuracy to measure the magnitude of this value, the larger this value, the lower the compensation accuracy. Further improving the compensation accuracy can be achieved by shrinking the clk. There are two ways to optimize the clk, either by reducing the size of the transistors inside the D-triggers or using a more integrated process [18,19].

**Figure 7:**
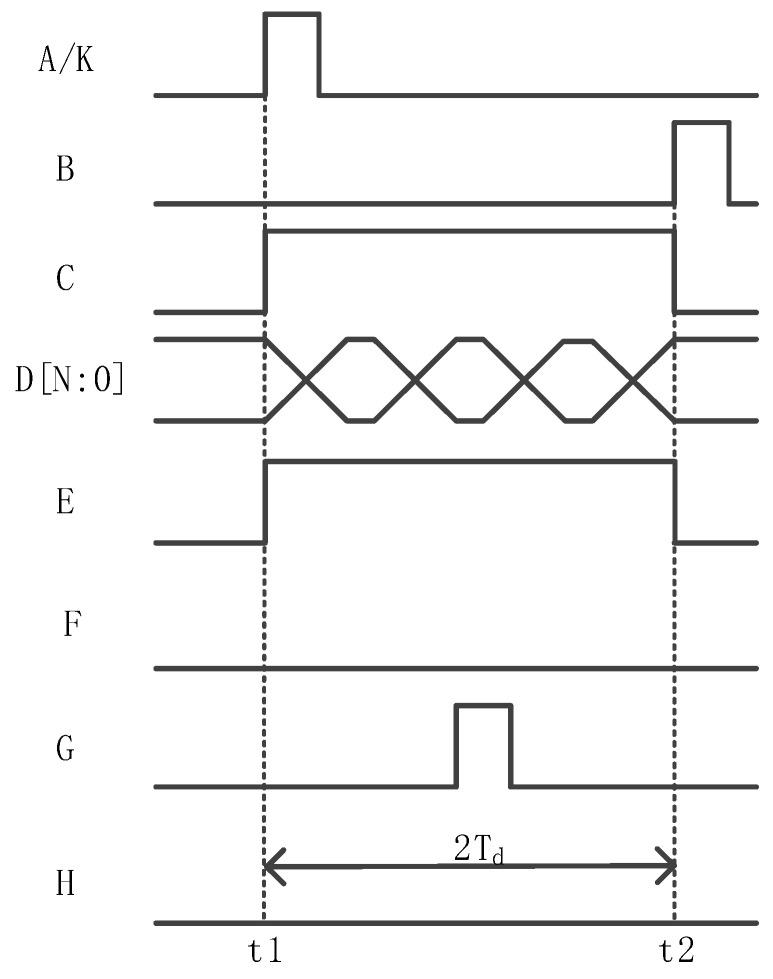
Delay detection working timing.

The working sequence of the delay time detection function is shown in Figure 7. The signals at the A–H and K terminals are 0 at the initial time. F and H are always kept at 0 during the delay time detection phase.

Firstly, at time t1, the timing module outputs logic 1 from the E terminal to turn on the counter and enable control. At the same time, the timing control module outputs pulse signal A from the K output terminal and reaches the A input terminal of the logic operation circuit first. Because the distance from the K end to the A end is very short, the signal at the K end and the signal at the A end are almost in phase. At this point, the logic operation circuit detects the rising edge of the signal at the end of A, outputs the high level from C, and controls the counter to start counting.

Then, when the pulse signal output from the sequencer K reaches the input terminal G of the row driver module on the right through buffer chain 2, the pulse signal returns from buffer chain 3 and reaches the B input terminal of the logic operation circuit at time t2. As soon as the logic operation detects the rising edge of terminal B, it outputs the low level from the output terminal C and pauses the counter counting. The count (D[N:0]) is the total delay of buffer chains 2 and 3. Since buffer chains 2 and 3 have the same delay, half of the counter output (D[N:1]) is the delay value of buffer chain 2. This completes the delay detection process.

Finally, half of the count value (D[N:1]) is configured for the delay time of the compensation circuit, and the compensation circuit will generate a corresponding delay according to the configured count value.

After that, the output signal of the sequencer reaches the input terminal F of the left row driver module through the compensation circuit and reaches the input terminal G of the right row driver module through buffer chain 2. The delay time of the compensation circuit is equal to the delay time of buffer chain 2, so the signals at points F and G can maintain a high degree of synchronization.

The delay time of a buffer chain composed of n-stage buffers is

(2) $$T_{d}\;=\;\ln(2){\mathrm{nR}}_{\mathrm{eq}}C_{\mathrm{eq}}$$

where C_eq_ is the parasitic capacitance, and R_eq_ is the parasitic resistance.

In the digital back-end design, since these buffers are generated using RTL code and the number of parallel buffers is 1, the buffer size and supply voltage are fixed. So, the delay time T_d_ is only related to the parasitic on the wire and the load capacity of the supply. The load capacity of the power supply here refers not only to the load capacity of the external regulator voltage but also to the parasitic resistance introduced by the long trace of the power supply in the layout, which limits the charging and discharging ability of the parasitic capacitor and then leads to an increase in the delay [20].

As shown in Figure 5, the signal sent by the timing control module reaches the row driving module on the left of the pixel array after a delay of T_d1_ time through the shorter buffer chain 1 and reaches the row driving module on the right of the pixel array after a delay of T_d2_ time through the longer buffer chain 2, and the delay time T_d2_ is greater than the delay time T_d1_. The delay difference between T_d1_ and T_d2_ is expressed as

(3) $$\Delta T\;=\;|T_{d1}\;-T_{d2}|$$

**Figure 8:**
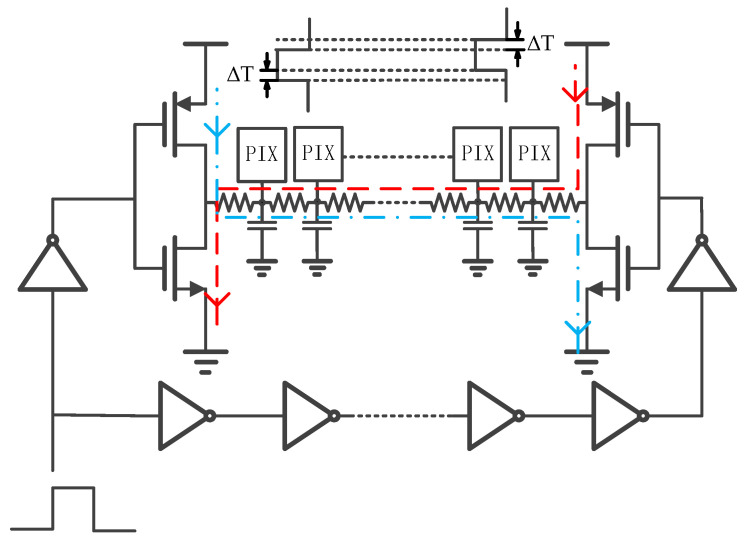
Current flooding between two row drives caused by a delay time difference.

ΔT directly leads to the non-synchronization of the output signals of the row driver modules on both sides, which will reduce the settling speed of the row bus signal in the pixel array. If ΔT is too large, it may cause the DC shoot-through phenomenon in the left and right row driver modules, as shown by the red and blue lines in Figure 8, which will increase the additional power consumption.

**Figure 9:**
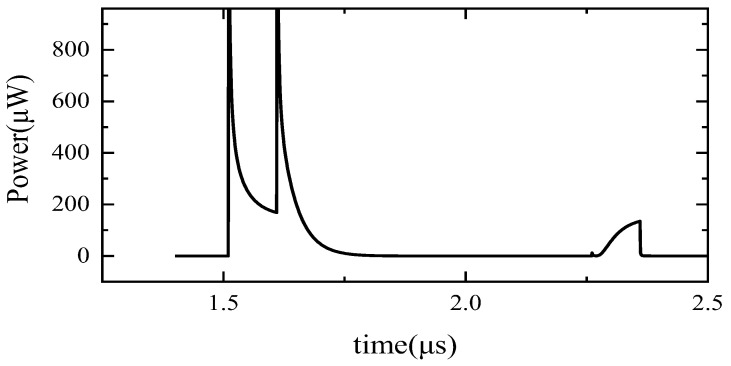
The DC shoot-through phenomenon when T is large enough.

When the TX signals from the left and right inputs are not synchronized, as shown in Figure 1, a transient power consumption of 120 μW is caused, as shown in Figure 9. Designers should avoid this situation.

**Figure 10:**
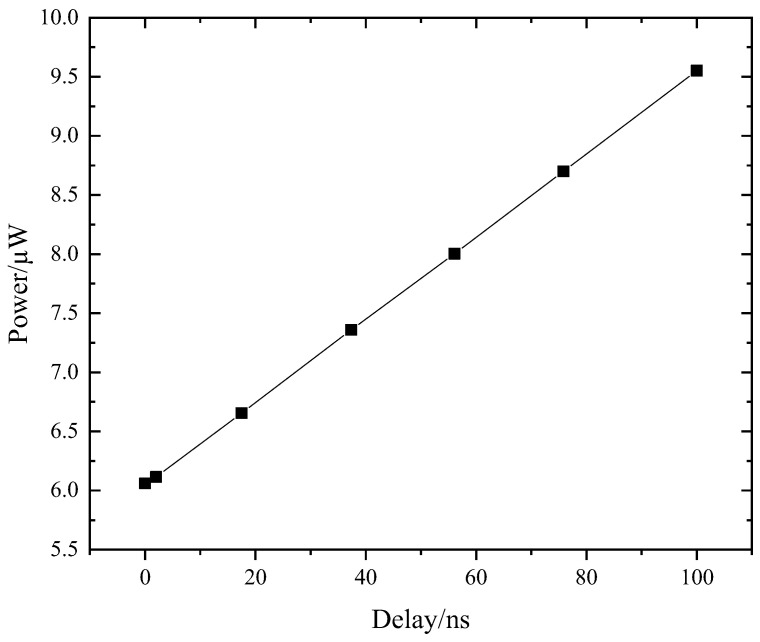
Increased signal inconsistency will increase row driver power consumption.

The specific measure of compensation accuracy is related to the feature size of the process used. Typically, the smaller the feature size of the process used, the higher the compensation accuracy. Figure 10 demonstrates that using a domestic 55 nm process, the time difference between the inconsistencies in the signals received by the left and right row drivers is linearly related to the average row driver power consumption. Therefore, considering the power consumption, we want the left and right row drivers to receive the same signal as much as possible. The term “the delay of the right row driver input signal from the left row driver input signal” is briefly described as the “delay”.

## 4. Self-Calibration Method Based on Delay Detection and Accurate Compensation

**Figure 11:**
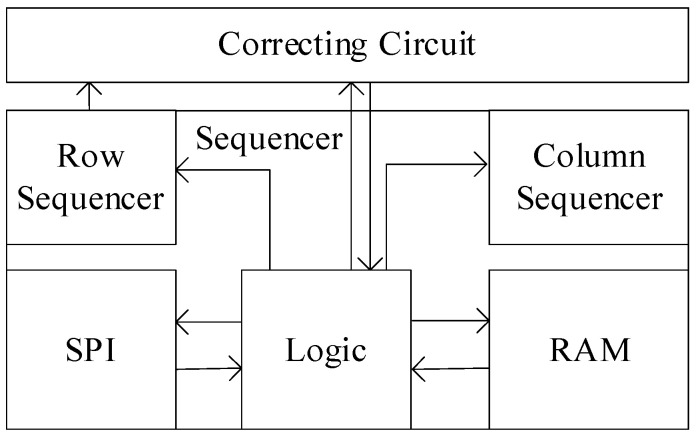
The structure of the sequencer in a CIS.

The design of the sequencer is divided into three main categories, one for generating timing control signals for the pixels, a second for the selection of the sensor’s functional mode, and a third for the design of the on-chip registers. These designs are implemented at the physical level through RTL code and back-end automated device placement and routing. The sequencer structure is shown in Figure 11. SPI is used to communicate with the FPGA, RAM is used to store the configuration information of each module, and logic issues instructions to the row sequencer and column sequencer according to the configuration information. The row sequencer generates the periodic signals required by the row logic, and the column sequencer is used to generate the timing of the column readout circuit. The proposed correction circuit is also controlled by logic.

**Figure 12:**
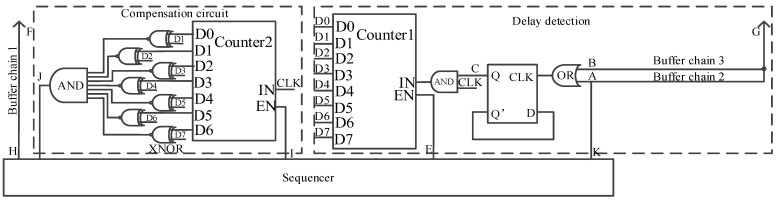
The specific implementation circuit of adaptive correction.

The specific implementation circuit for the delay detection and compensation technology in Figure 5 is shown in Figure 12. OR, D flip-flop, and AND: these three constitute the logic operation circuit. The enable EN of counter 1 is controlled by a sequencer.

Counter 2, 7 XNORs, and 7 input AND constitute the compensation circuit. The enabling of counter 2 is controlled by the sequencer, and the input of 7 XNORs is connected to the high 7 bit D[7:1] of counter 1, which is the delay time of buffer chain 2.

**Figure 13:**
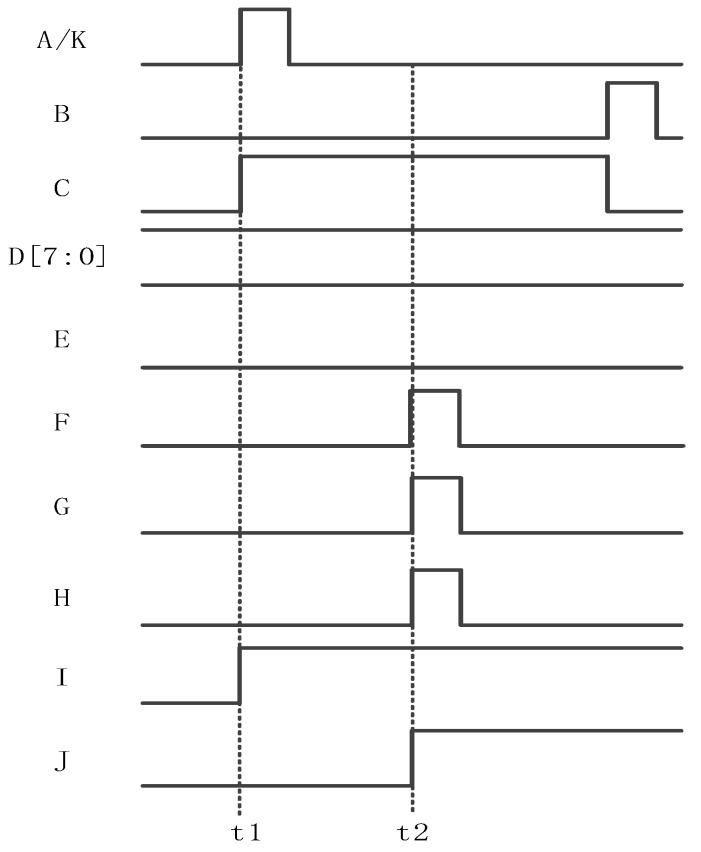
The realized waveform of the bilateral synchrony drive.

After the circuit completes the delay detection process according to the timing sequence shown in Figure 7, the EN of counter 1 is set low so that the count value D[7:0] is maintained. The high 7 bits D[7:1] of counter 1 are, respectively, input into the input terminals of the 7 XNORs in the delay detection circuit to complete the delay time configuration of the compensation circuit. The specific realized waveform of the bilateral synchronization driver is shown in Figure 13, where the signals from A–J are all logic 0 at the initial time.

Firstly, at time t1, the sequencer emits a timing signal from the K terminal and transmits it to the G terminal, the input of the right-side row driver. At the same time, the I side outputs logic 1, the counter is enabled to be valid, and counter 2 starts counting. Second, at time t2, when the count value of counter 2 is equal to the value of the higher 7 bits of counter 1, 7 inputs AND outputs logic 1 to the sequencer. After the sequencer receives logic 1, it outputs the timing signal from the H terminal and arrives at the left row driving input terminal F. At this time, the signal output from the K terminal at time t1 also arrives at the G terminal, realizing the signal synchronization between the F terminal and the G terminal.

In this way, the synchronization of the signal of the bilateral row driver module can be greatly improved, and the pixel array can obtain a synchronous driving signal from the left and right sides at the same time, which can effectively prevent current flooding. The circuit can start to perform delay detection after the chip is powered on. When the CIS is hot due to continuous operation, to ensure synchronization, one delay detection can also be performed before the exposure starts to achieve a working state of correction.

## 5. Simulation Results and Analysis of Delay Detection and Accurate Compensation Technology

**Figure 14:**
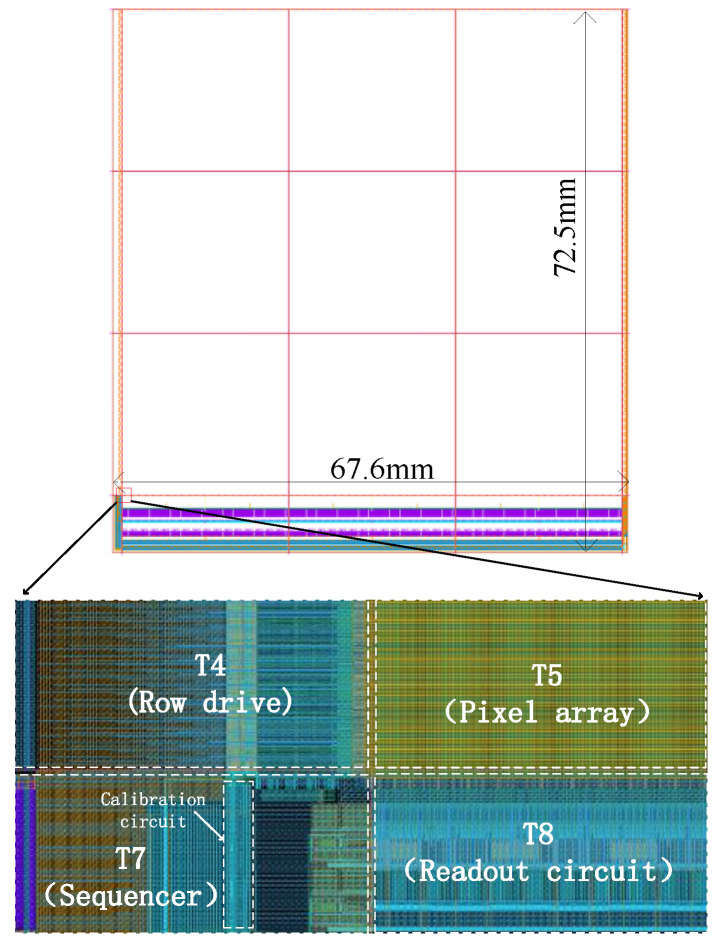
The overall layout and the specific location of the calibration circuit.

The design is based on 55 nm CMOS image sensor technology, and the main clock is a 500 MHz fixed working frequency; in the case of different wire widths and different power supply band load capacities, the proposed non-synchronization solution is verified using simulations. The layout of the 12,288 × 12,288 CIS is shown in Figure 14, and the proposed correction circuit is located in the T7 stitching block.

**Figure 15:**
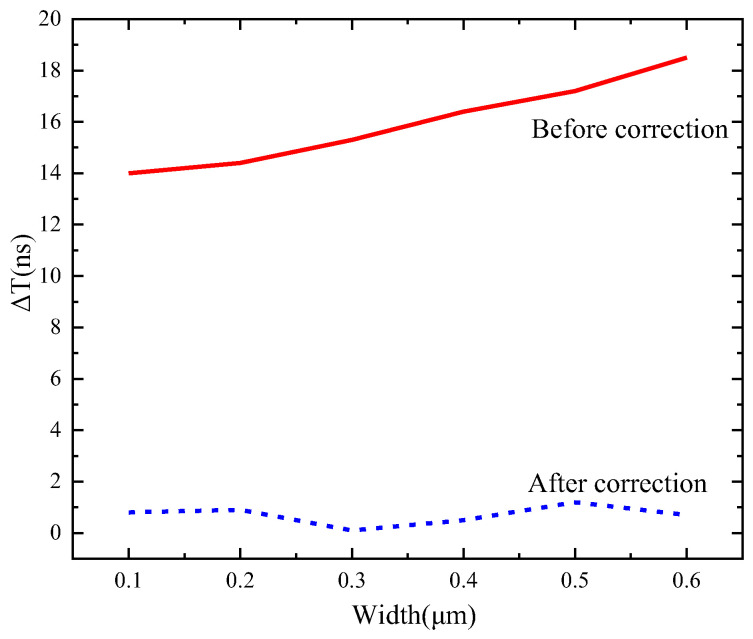
Relationship between wire width and delay time difference before and after correction.

To verify the influence of parasitic effects on the performance of the correction circuit when the power supply is ideal, buffer chains with different wire widths are designed and post-layout simulated. The distance between the output port of the timing control module and the input terminal of the right row driver is about 73 mm. When the power is ideal, a buffer is inserted at 1 mm intervals, and the relationship between the wire width and the delay time difference (ΔT) is shown in Figure 15. The post-layout simulation data show that the delay time difference is higher when the wire width is wider. The delay time τ is determined by RC, and when the wire width increases, R decreases and C increases, leading to an increase in the value of τ, thus causing ΔT to increase with increasing wire width.

**Figure 16:**
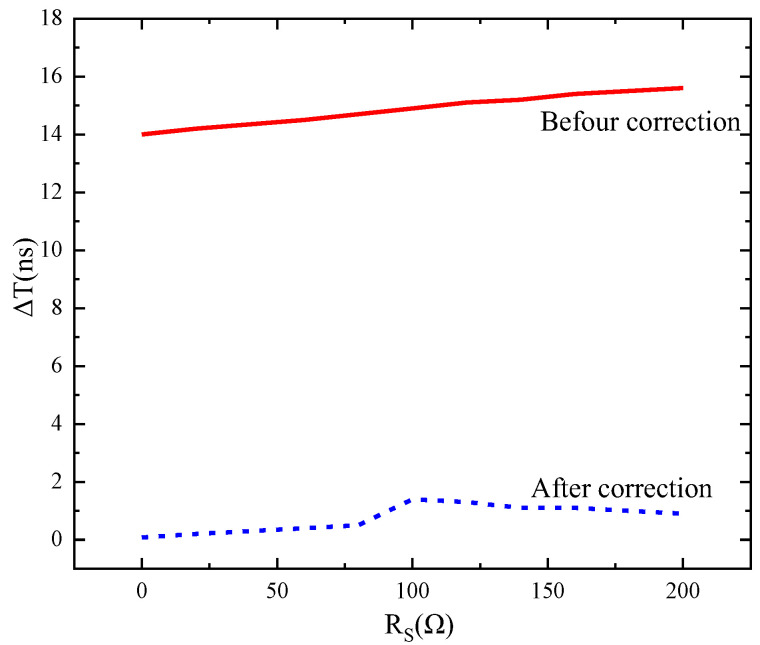
Relationship between delay time difference and power supply internal resistance before and after correction.

Limited by the level of the manufacturing process, the minimum wire width is 0.1 μm, and the delay time is affected by the driving capability of the power supply in the case of a buffer interval of 1 mm. The measure of the driving ability of the power supply is the internal resistance of the power supply. A strong power supply driving ability means that the internal resistance (Rs) of the power supply is close to 0. The post-layout simulation results are shown in Figure 16. When the internal resistance of the power supply is higher, the delay time difference between the left and right sides increases linearly. When the internal resistance of the power supply is large, the charging and discharging speed of the parasitic capacitor is slowed down, and the delay time is increased.

After using the correction technique, the time difference of the bilateral row drive is reduced from 14–25.2 ns to 0.03–1.7 ns in the power supply internal resistance interval from 0 Ω to 600 Ω. After correction, the time difference ΔT between the arrival of the signal to the two sides is less than 1 clock cycle and does not vary significantly with the internal resistance of the power supply.

**Figure 17:**
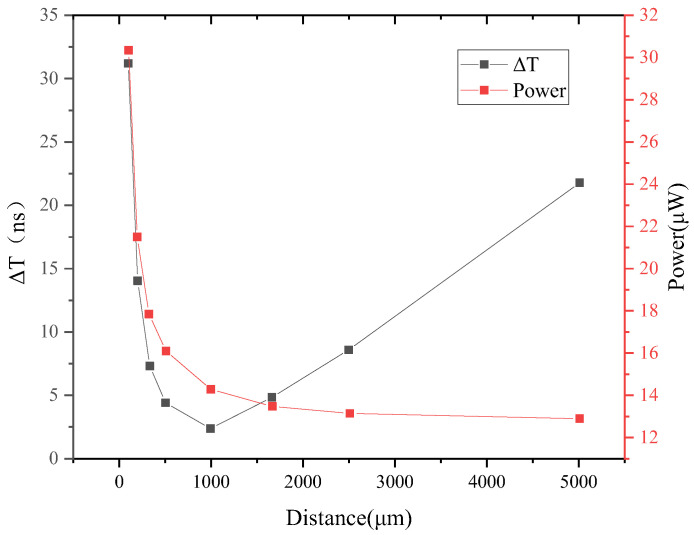
Effect of buffer spacing on delay time difference.

In the case of an ideal power supply, a minimum line width of 0.1 μm is used, and the effect of the buffer spacing on the delay time difference is simulated post layout, and the results are shown in Figure 17. With an increase in buffer spacing, the delay time difference first decreases and then increases, and the delay time difference is the smallest when the spacing is about 1 mm. The power consumption decreases as the buffer spacing increases.

**Figure 18:**
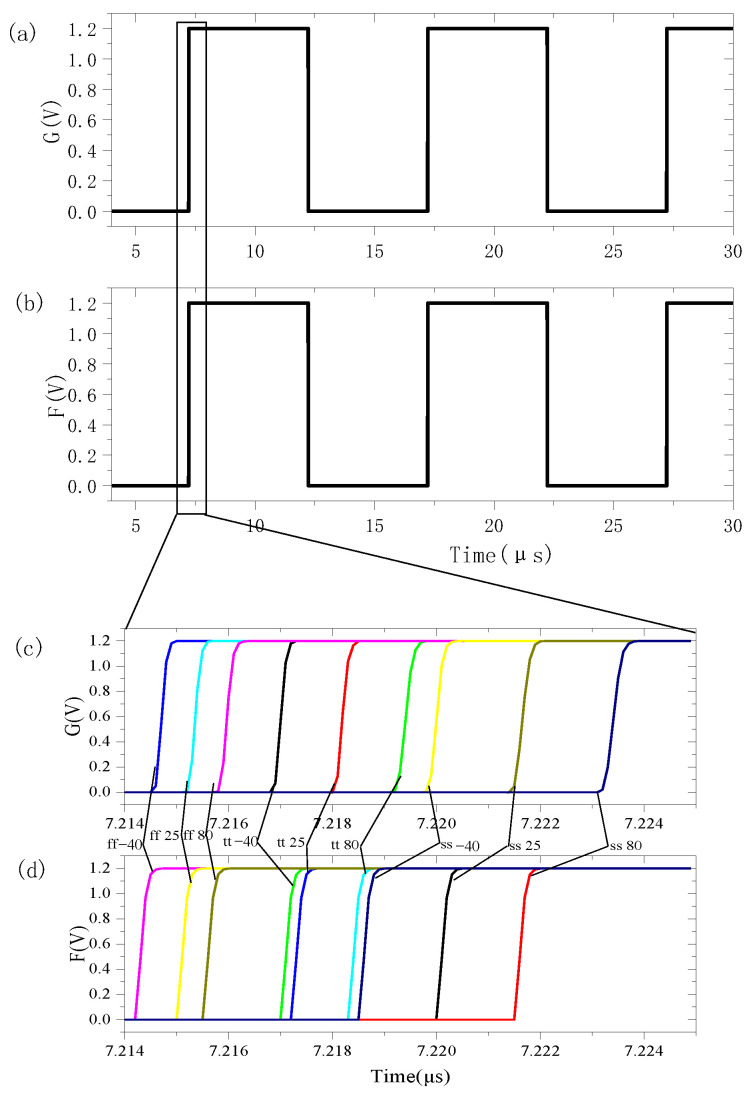
The corrected TX PVT waveforms at the F and G ports.

To further verify the reliability of the calibration circuit, the performance of the circuit is simulated in different corners and different temperature environments. Taking the TX signal as an example, the corrected signal at the F and G ports are shown in Figure 18a,b. The amplified waves of the rising edge of TX signals during PVT simulation is shown in Figure 18c,d. The PVT results show high synchronization of the TX signals at the F and G ports.

**Figure 19:**
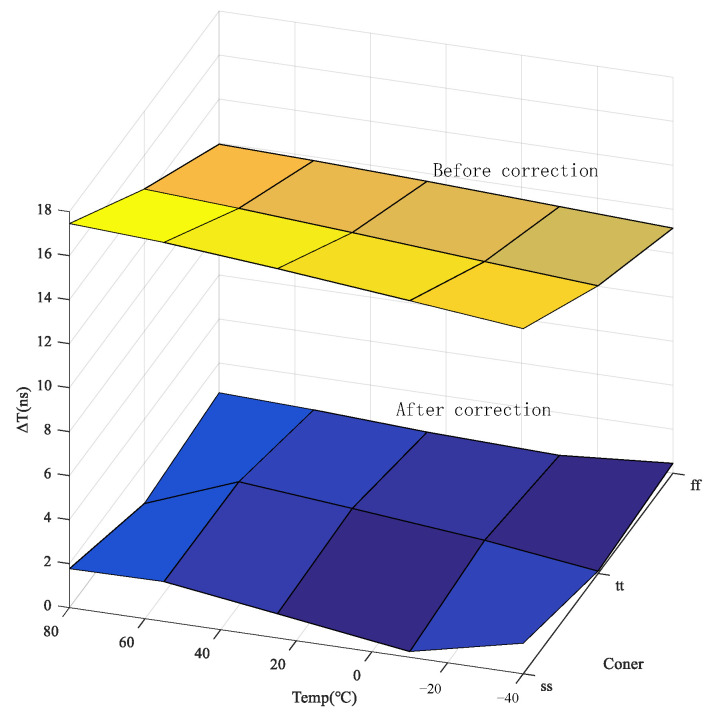
Simulation results before and after correction for different temperatures and corners.

The simulation results before and after correction are plotted in a 3D form and shown in Figure 19. The corrected delay difference is stable between 0.17 ns and 1.81 ns and almost does not change significantly with the changes in temperature and corner. Before the correction, the delay difference is between 11.1 ns and 17.5 ns in the temperature range, and the delay difference changes approximately linearly with the changes in temperature and corner.

According to the above results, when the size of the buffer is fixed, the delay time has a great relationship with the wire width of the metal wire, the driving ability of the power supply, the spacing of the buffer, the temperature, and the process corner. From the circuit design, the delay time of the buffer chain can be reduced by reducing the wire width, improving the driving ability of the power supply, and maintaining appropriate buffer spacing.

**Figure 20:**
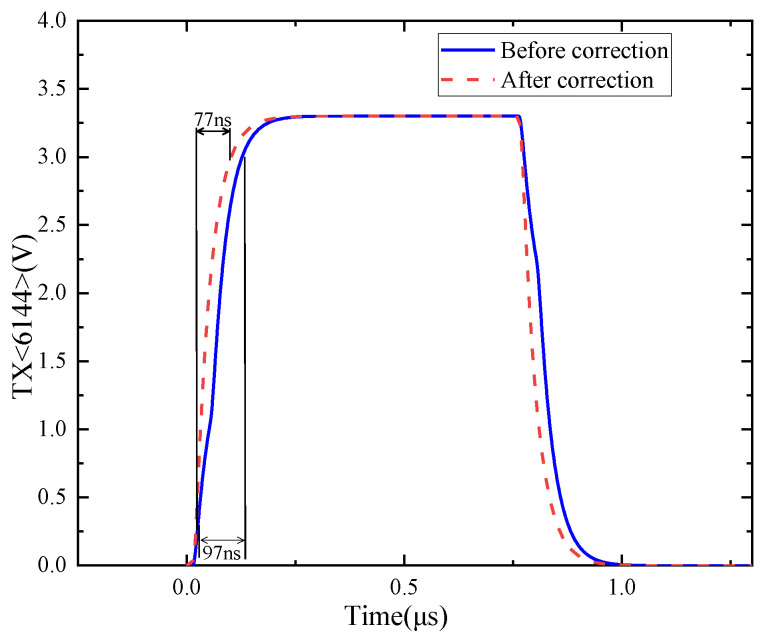
Comparison of row driver signal rise time in the middle column before and after correction.

After applying the correction technique, the rise time of the pixel driving signal in the middle column is reduced from 97 ns to 77 ns, as shown in Figure 20. When designing the correction circuit, the accuracy of the correction circuit can be determined according to the CIS reading row time and the requirements of the driving signal settling time.

Through the above simulation results in the case of different wire widths, power supply internal resistances, temperatures, and corners, it can be concluded that the adaptive correction technology based on delay detection and accurate compensation has high reliability. As shown in the comparison of specifications in Table 1, the non-synchronization after the correction is less than 2 ns, which is slightly worse than the non-synchronization of less than 1 ns achieved via the PLL correction technique adopted in Reference [15]. However, the method proposed in this paper uses a digital circuit with very low power consumption. The additional power consumption introduced by the calibration circuit is less than 3 μW. With the help of correction techniques, this design achieves 10 FPS. The advanced 3D process CIS achieves the same frame rate [17], but this design has a much larger pixel size.

## 6. Conclusions

This paper analyzes the problem of bilateral row drive signal non-synchronization encountered in the design process of stitching large-array CMOS image sensors and analyzes the specific reason for the problem in detail. To solve this problem, an adaptive correction technique based on time delay detection and accurate compensation is used in the design. Based on a 55 nm stitching process, the synchronization of bilateral row driver output signals is realized in a CMOS image sensor with a 150 M pixel scale. At a clock frequency of 500 MHz, the row clock is 125 kHz. Post-layout simulation results show that the application of the correction circuit reduces the non-synchronization of the drive signals from more than 17.5 ns to less than 2 ns, and the synchronization is improved by more than 8.7 times, while the power consumption is less than 3 μW. It ensures that the frame rate of the mega-array image sensor with a scale of 100 million pixels can reach more than 10 FPS.

## Figures and Tables

**Table 1:** Comparison of specifications.

Work	[8]	[15]	[17]	This Work
Process	55 nm, stitching	55 nm, stitching	65 nm, 28 nm	55 nm, stitching
Pixel size (μm)	5.67	7.5	0.7	5.5
Pixel array (H) × (V)	5644 × 7954	15,360 × 15,360	12,000 × 9000	12,288 × 12,288
Layout area (H) × (V) (mm)	38.8 × 28.3	118 × 125	-	72.5 ×67.6
FPS	2.5	10	10	10
Correction techniques	-	DLL	-	Delay detection and compensation
Correction accuracy (ns)	-	<1	-	<2
Correcting circuit power (W)	-	>5 m	-	<3 μ

## Data Availability

The data presented in this study are available on request from the corresponding author. The data are not publicly available due to privacy.

## References

[1] Kim Y., Choi W., Park D., Jeoung H., Kim B., Oh Y., Oh S., Park B., Kim E., Lee Y. A 1/2.8-inch 24Mpixel CMOS image sensor with 0.9 μm unit pixels separated by full-depth deep-trench isolation. Proceedings of the 2018 IEEE International Solid-State Circuits Conference-(ISSCC).

[2] Kim H., Park J., Joe I., Kwon D., Kim J.H., Cho D., Lee T., Lee C., Park H., Hong S. 5.6 A 1/2.65in 44Mpixel CMOS Image Sensor with 0.7 µm Pixels Fabricated in Advanced Full-Depth Deep-Trench Isolation Technology. Proceedings of the 2020 IEEE International Solid-State Circuits Conference-(ISSCC).

[3] Hsu T.H., Chen Y.K., Chiu M.Y., Chen G.C., Liu R.S., Lo C.C., Tang K.T., Chang M.F., Hsieh C.C. (2021). 5.9 A 0.8V Multimode Vision Sensor for Motion and Saliency Detection with Ping-Pong PWM Pixel. IEEE J. Solid-State Circuits.

[4] Song K., Kim D., Kim J., Yoo J., Keum W., Rieh J.S. (2022). A Scalable 300-GHz Multichip Stitched CMOS Detector Array. IEEE Trans. Microw. Theory Tech..

[5] Xu J., Li W., Nie K., Han L., Zhao X. (2019). A Method to Reduce the Effect on Image Quality Caused by Resistance of Column Bus. IEEE Trans. Very Large Scale Integr. (VLSI) Syst..

[6] Gao J., Zhang D., Nie K., Xu J. (2019). Analysis and Optimization design of the column bus parasitic effects on large-array CMOS image sensor. Microelectron. J..

[7] Bogaerts J., Lafaille R., Borremans M., Guo J., Ceulemans B., Meynants G., Sarhangnejad N., Arsinte G., Statescu V., van der Groen S. 6.3 105 × 65 mm2 391Mpixel CMOS image sensor with >78 dB dynamic range for airborne mapping applications. Proceedings of the 2016 IEEE International Solid-State Circuits Conference (ISSCC).

[8] Zhu J., Liu D., Zhang W., Wang Q., Li W., Chen L., Zhao Y. (2016). Systematic experimental study on stitching techniques of CMOS image sensors. IEICE Electron. Express.

[9] Gao J., Zhang T., Nie K., Xu J. (2021). Design of Timing Driven Circuit for Ultra Large Array CMOS Image Sensor. J. Tianjin Univ..

[10] Song Y., Li P., Liu Z., Xi W., Yao H., Zheng D., Huang K. Buffer Reduction for Congestion Control during Timing Optimization. Proceedings of the 2022 IEEE 2nd International Conference on Power, Electronics and Computer Applications (ICPECA).

[11] Athreya S., Hedayati H., Kazemkhani S., Chen Y., Vats S., Scott M.D., Zeydel B., Keller P., Wang J., Avula B. Clock Synchronous Reset and Skew Calibration of 65GS/s ADCs in A Multi-Lane Coherent Receiver. Proceedings of the ESSCIRC 2018-IEEE 44th European Solid State Circuits Conference (ESSCIRC).

[12] Yasutomi K., Okura Y., Kagawa K., Kawahito S. (2019). A Sub-100 μm-Range-Resolution Time-of-Flight Range Image Sensor with Three-Tap Lock-In Pixels, Non-Overlapping Gate Clock, and Reference Plane Sampling. IEEE J. Solid-State Circuits.

[13] Miao L., Yasutomi K., Imanishi S., Kawahito S. (2015). A Column-Parallel Clock Skew Self-Calibration Circuit for Time-Resolved CMOS Image Sensors. IEICE Electron. Express.

[14] El-Chammas M., Murmann B. (2011). A 12-GS/s 81-mW 5-bit Time-Interleaved Flash ADC with Background Timing Skew Calibration. IEEE J. Solid-State Circuits.

[15] Guo Z.J., Yu N.M., Wu L.S. (2020). A synchronous driving approach based on adaptive delay phase-locked loop for stitching CMOS image sensor. IEICE Electron. Express.

[16] Totsuka H., Tsuboi T., Muto T., Yoshida D., Matsuno Y., Ohmura M., Takahashi H., Sakurai K., Ichikawa T., Yuzurihara H. 6.4 An APS-H-Size 250Mpixel CMOS image sensor using column single-slope ADCs with dual-gain amplifiers. Proceedings of the 2016 IEEE International Solid-State Circuits Conference (ISSCC).

[17] Jun J., Seo H., Kwon H., Lee J., Yoon B., Lee Y., Kim Y., Joo W., Lee J., Koh K. A 0.7 μm-Pitch 108 Mpixel Nonacell-Based CMOS Image Sensor with Decision-Feedback Technique. Proceedings of the 2022 IEEE International Symposium on Circuits and Systems (ISCAS).

[18] Sato Y., Yamada T., Nishimura K., Yamasaki M., Murakami M., Urabe K., Eriguchi K. Evaluation of Plasma-Induced Stochastic Damage Creation in the Lateral Direction using pn Junction Structures. Proceedings of the 2021 20th International Workshop on Junction Technology (IWJT).

[19] Su C., Guo Z.J., Li C., Liu S., Cao X., Han X. An Acceleration Technology in CMOS Image Sensor Readout Circuit. Proceedings of the 2020 IEEE 15th International Conference on Solid-State & Integrated Circuit Technology (ICSICT).

[20] Guo Z., Cheng X., Xu R., Su C., Li C., Wang B., Guo Y., Wang Y. (2023). A 1Gpixel 10FPS CMOS image sensor using pixel array high-speed readout technology. Integr. VLSI J..

